# Conservation and Divergence of *E(z)* Genes in Green Plants

**DOI:** 10.3390/plants14223444

**Published:** 2025-11-11

**Authors:** Xiaolong Gan, Zihua Chen, Liangsheng Zhang, Xiaojun Chang

**Affiliations:** 1 College of Agriculture and Biotechnology, Zhejiang University, Hangzhou 310058, China; ganxiaolong_93@163.com; 2 Fujian Provincial Key Laboratory of Haixia Applied Plant Systems Biology, Center for Genomics and Biotechnology, College of Life Science, Fujian Agriculture and Forestry University, Fuzhou 350002, China

**Keywords:** PcG proteins, *E(z)* genes, green plants, conservation and divergence, positive selection

## Abstract

Polycomb Group (PcG) proteins, particularly *E(z)* (Enhancer of Zeste) genes, play essential roles in transcriptional repression and developmental regulation. To investigate their evolutionary history, we conducted a comprehensive comparative genomic analysis of *E(z)* homologs across green plants. Phylogenetic analysis revealed that *E(z)* genes are highly conserved, predominantly occurring as single copies in green algae and early land plants. In seed plants, however, *E(z)* homologs diverged into two major clades, *CLF* and *SWN*, likely originating from an ancient duplication predating seed plant diversification. Conserved domain and motif analyses showed that while all E(z) proteins contain the hallmark SET domain, certain lineages also harbor CXC and SANT domains. Moreover, lineage-specific motif divergence was observed, suggesting functional diversification. In angiosperms, further duplications shaped the *SWN* lineage: in Brassicaceae, *SWN* genes split into *SWN* and *MEA* subclades, whereas in Fabaceae, *SWN* genes diverged into *SWN1* and *SWN2*. Structural comparisons revealed that both Brassicaceae *MEA* and Fabaceae *SWN2* proteins independently lost approximately 200 amino acids in the central region, indicating convergent structural modifications. Molecular evolutionary analysis showed that Fabaceae *SWN1* genes are under purifying selection, consistent with retention of ancestral functions, whereas *SWN2* genes experienced strong positive selection, implying functional innovation. Expression profiling of soybean *E(z)* genes further supported this scenario: *SWN1* is broadly expressed across tissues, while *SWN2* expression is restricted to the heart-shaped embryo. This pattern mirrors Arabidopsis *MEA*, suggesting that Fabaceae *SWN2* may have evolved imprinted gene functions critical for seed development. Together, our results highlight the evolutionary conservation of *E(z)* genes in plants and reveal how gene duplication and lineage-specific divergence have driven functional specialization, particularly in Fabaceae *SWN2*.

## 1. Introduction

The Polycomb group (PcG) proteins are key epigenetic regulators of gene expression in both animals and plants [1,2,3]. They methylate histones and modify chromatin [1,4], thereby maintaining allele-specific silencing of certain imprinted genes [5,6]. PcG proteins assemble into multiple protein complexes, such as Polycomb Repressive Complex 1 (PRC1) and Polycomb Repressive Complex 2 (PRC2) [7]. Early research demonstrated that PcG proteins are conserved between plants and animals [7,8,9,10]. In animals, PcG proteins are involved in maintaining pluripotency and preventing premature cell differentiation [11]. In plants, the PcG complexes were first identified in 1997, and since then, a functional PcG system has been found in all extent multicellular plants and in several unicellular algae [12]. Plant PcG proteins participate in multiple developmental processes, including seed formation, flower development and the vernalization response [13]. They also promote cell differentiation by repressing embryonic developmental programs [14].

The E(z) (Enhancer of zeste) protein is a core component of PRC2 and belongs to the SET [Su(var)3-9; E(z); Trithorax] domain family [15] in *Drosophila*. It catalyzes the methylation the lysine 27 of histone H3 (H3K27) [16] and plays key roles in establishing and maintaining transcriptional repression [17]. In vertebrates, EZH1 and EZH2 are core PRC2 components that function as H3K27 methyltransferases (H3K27me3) [18,19]. In *A. thaliana*, three E(z) homologs, CURLY LEAF (CLF), MEDEA (MEA), and SWINGER (SWN), have been identified [20,21,22]. Previous studies have shown that these genes control seed development, flower development and vernalization [23]. An E(z) homolog (CLF) has also been identified in the moss *Physcomitrella patens* [24], and E(z) homologs have been reported in green algae as well [12].

However, a systematic study of this gene family, particularly in the context of seed plant evolution, is still lacking. In this study, we address this gap by reconstructing the evolutionary history of *E(z)* genes across green plants. Our findings reveal that *E(z)* homologs are distributed throughout the green plant lineage and that gene duplication events have driven their diversification and functional specialization.

## 2. Results

### 2.1. Identification of E(z) Genes in Green Plants

**Figure 1:**
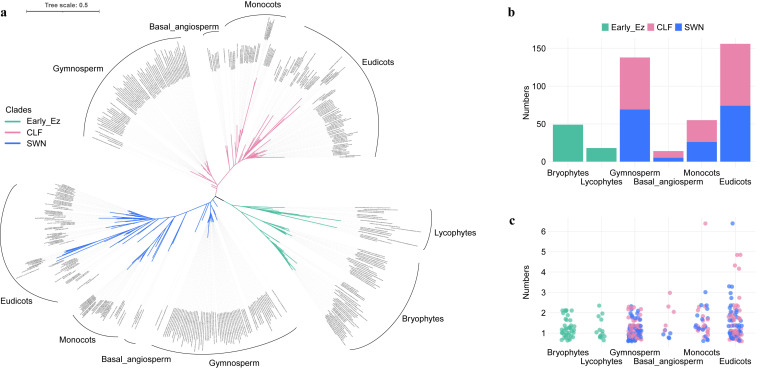
The phylogenetic analysis of *E(z)* genes and the gene numbers in different green plant groups. (**a**), *E(z)* gene tree in green plants. (**b**), the total *E(z)* gene numbers in 6 plant groups. (**c**), *Early_E(z)*, *CLF* and *SWN* gene numbers of each species in each group. Different colored branches in the phylogenetic tree represent different plant groups. The *E(z)* genes in non-seed plants are designed as Early E(z).

To comprehensively elucidate the origin and evolutionary trajectory of plant *E(z)* homolog genes, we obtained 431 E(z) protein sequences for phylogenetic analysis: 51 eudicots (157 sequences), 18 monocots (55 sequences), 5 basal angiosperm (14 sequences), 66 gymnosperms (138 sequences), 14 lycophytes (18 sequences), and 39 bryophytes (49 sequences) (Supplementary Tables S1–S7). These sequences were retrieved from genomic databases (232 sequences) and the transcriptome database OneKP (199 sequences). The topological structure of the phylogenetic tree reveals that bryophytes and lycophytes, as early green plants, distinctly cluster within a large branch, which we refer to as the early Ez branch. The remaining *E(z)* genes are clearly divided into two major clades. Based on marker genes from *A. thaliana*, we classified these into the *SWN* clade and the *CLF* clade (Figure 1a). Within these two clades, the phylogenetic tree is arranged in the order of gymnosperms, basal angiosperms, monocots, and eudicots, a result that aligns with the existing classification structure of green plants (Figure 1a). We further examined the distribution of *E(z)* genes in each species of 6 major green plant groups (Figure 1b). In all lineages of early land plants, *E(z)* genes were present in single or low copy numbers, whereas in seed plants they occurred in multiple copies, especially in angiosperms (basal angiosperms, monocots and eudicots) (Figure 1b,c), suggesting that copy number expansion took place at later evolutionary stages.

### 2.2. The Duplication and Divergence of E(z) Genes in Seed Plants

**Figure 2:**
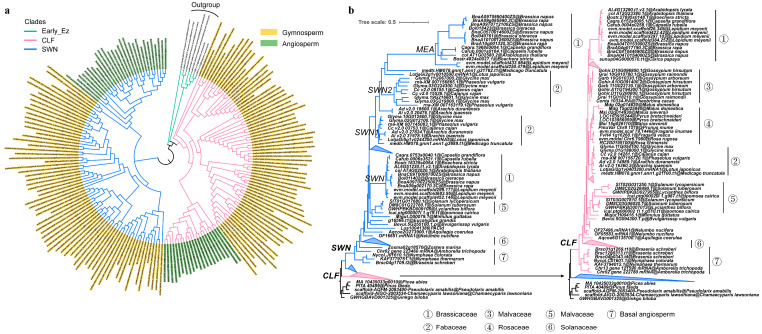
Phylogenetic tree of *E(z)* genes in seed plants and in angiosperms. (**a**), Phylogenetic tree of *E(z)* genes in seed plants. (**b**), Phylogenetic tree of *E(z)* genes in Angiosperms. The seven numbers represent seven distinct families or groups. The clade *SWN*, *CLF*, and *Early_E(z)* clades were colored in blude, pink, and green, respectively. Within the Brassicaceae, the *SWN* is further divided into two subclades: the *SWN* clade and the *MEA* clade. In the Fabaceae family, the *SWN* clade is also divided into two subclades: *SWN1* and *SWN2*.

Given the expansion of *E(z)* genes in seed plants, it is important to explore their diversity. To this end, we reconstructed phylogenetic trees using protein sequences from 13 angiosperms, 65 gymnosperms, and 4 outgroups (Supplementary Table S8). The results confirmed the split into two clades, *SWN* and *CLF*, named after the *A. thaliana* marker genes *SWN* (*AT4G02020*) and *CLF* (*AT2G23380*). Each clade includes both gymnosperm and, notably, multiple angiosperm genes (Figure 2a and Supplementary Table S7), suggesting that the two clades originated from a gene duplication event that occurred prior to the divergence of seed plants. To further distinguish the two groups, we conducted a motif analysis (Supplementary Figure S1). A clear difference emerged: the *CLF* clade was relatively more conserved, consistently retaining motifs such as 14 and 19, whereas most *SWN* clade lacked one or both. This motif-based pattern mirrors the phylogenetic tree topology, reinforcing the conclusion that *CLF* and *SWN* represent distinct evolutionary lineages within seed plants. Selection analysis based on both protein and coding sequences supported this view. The *SWN* clade (ω = 0.51), where ω represents the ratio of nonsynonymous to synonymous substitution rates, was under stronger positive selection than the *CLF* clade (ω = 0.06) (Supplementary Figure S2). Taken together, these results indicate that *CLF* represents the more functionally conserved lineage, whereas *SWN* has undergone greater divergence since their ancient split.

### 2.3. The Divergence of E(z) Genes in Angiosperms

Given the clear diversification of the *E(z)* gene family in seed plants and the importance of epigenetic modification in angiosperm growth, development, and flowering [25], we further examined the copy number and phylogenetic relationships of *E(z)* genes in angiosperms. Using 231 protein sequences from 75 species, we constructed a phylogenetic tree representing the major angiosperm lineages (Figure 2b and Supplementary Table S8), including eudicots (Brassicaceae, Fabaceae, Malvaceae, Solanaceae), monocots, and basal angiosperms (Nymphaeaceae, Amborellaceae). Five gymnosperm genomes were included as outgroups (Supplementary Table S8).

The phylogenetic analysis confirmed the presence of two major clades, *SWN* and *CLF* (Figure 2b). Gene expansion occurred in both clades. Within eudicots, multiple copies were observed in the *SWN* clade, particularly in Brassicaceae and Fabaceae (Figure 2b and Supplementary Table S8). In Brassicaceae, *Lepidium meyenii* and *Brassica napus* each have nine copies, while in Fabaceae, soybean exhibits the highest copy number with eight genes (Supplementary Table S8). The large-scale gene expansion in Brassicaceae is likely linked to a family-specific whole-genome duplication (WGD) event [26], which subsequently divided the SWN clade into two subclades (Figure 2b). Similarly, SWN genes in Fabaceae are grouped into SWN1 and SWN2 subclades, with clear synteny observed in soybean, indicating duplication via WGD (Supplementary Figure S3). Additional small-scale duplications were identified in several eudicots, including cotton (*Gossypium hirsutum*, 6 copies), apple (*Malus domestica*, 4), poplar (*Populus trichocarpa*, 4), willow (*Salix purpurea*, 5), and cactus (*Kalanchoe laxiflora*, 7).

In contrast, most angiosperms retained a single copy of *CLF* (Supplementary Tables S1 and S8). Exceptions with multiple copies were observed in several eudicots, such as *Capsicum pubescens* (5 copies), *Gossypium hirsutum* (4), and *Kalanchoe laxiflora* (5). Among monocots, small-scale duplications were less common and primarily detected in certain species, notably *Triticum aestivum* (Figure 2b and Supplementary Table S2). Collectively, these results demonstrate that the expansion of *E(z)* genes in angiosperms was primarily driven by whole-genome duplication.

### 2.4. Motif Patterns and Gene Structure of E(z) Genes in Fabaceae and Brassicaceae

**Figure 3:**
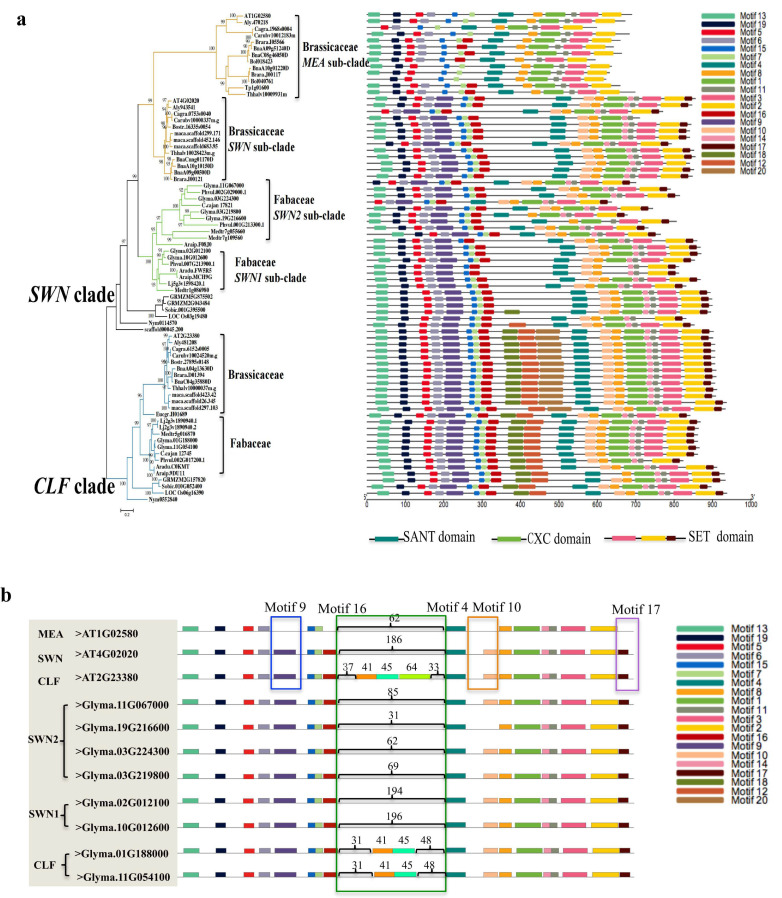
Conserved motif analysis in Fabaceae and Brassicaceae. (**a**), motif patterns of the *E(z)* genes in Fabaceae and Brassicaceae. (**b**), zoomed in motif pattern in *A. thaliana* and *G. max*. A total of 20 conserved motifs were identified, designated Motifs 1–20. The conserved motifs circled in four different colors represent conserved motifs that have diverged significantly.

To further investigate the sequence characteristics of *E(z)* genes in angiosperms, we focused on Fabaceae and Brassicaceae species, both of which have undergone notable expansions. Conserved motif analysis identified multiple shared motifs, including Motifs 1, 2, 3, 4, 5, 7, 8, 11, 13, 15, and 19, in both the *CLF* and *SWN* clades, reflecting overall sequence conservation (Figure 3a and Supplementary Figure S4). However, distinct differences were observed between clades and species. Compared with the *SWN* clade, the *CLF* clade specifically retained Motifs 12 and 18 (Figure 3a). In addition, Brassicaceae species uniquely evolved Motif 20, which is absent in Fabaceae (Figure 3). Amino acid composition analysis revealed that Motifs 12, 18, and 20 were enriched in the polar amino acid serine (Ser) and the basic amino acid lysine (Lys). By contrast, all SWN proteins lacked these three motifs (Figure 3a).

Using *A. thaliana* and *G. max* as examples, we found that the *SWN* subclade in Brassicaceae and the *SWN1* subclade in Fabaceae contain an insertion of ~200 non-conserved amino acids, whereas the *MEA* subclade in Brassicaceae and the *SWN2* subclade in Fabaceae carry a shorter insertion of ~30–80 amino acids (Figure 3b). Gene structure analysis suggested that these motif losses resulted from insertions or deletions within exon–intron structures (Supplementary Figures S5 and S6).

Alignment of conserved motif and domain boundaries further showed that Motif 9 overlapped with the N-terminal SANT domain, Motif 4 overlapped with the C-terminal SANT domain, Motif 1 overlapped with the CXC domain, and Motifs 2, 3, and 17 overlapped with the SET domain (Figure 3a and Supplementary Figure S7). This confirmed the correspondence between motifs and domains. Notably, the first SANT domain was absent in Brassicaceae, likely due to the loss of Motif 9, and was detected only in the Fabaceae *SWN* clades and in some members of the *CLF* clade (Supplementary Figure S7). These results suggest that both gene structure and protein function of *E(z)* homologs have undergone lineage-specific evolutionary changes in Brassicaceae and Fabaceae.

### 2.5. SWN2 Show Accelerated Amino Acid Substitution Rates and Evidence for Positive Selection in Fabaceae

**Figure 4:**
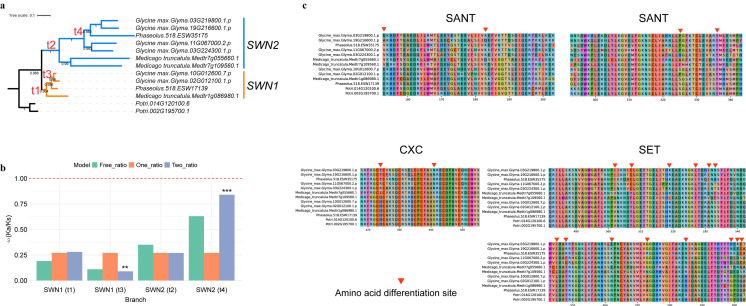
Selection analysis of *SWN1* and *SWN2* in Fabaceae. (**a**), Phylogenetic tree constructed by ML method, including species such as soybean, bean, alfalfa and poplar. (**b**), The Ka/Ks values for clades *SWN1* (t1), *SWN2* (t2), *SWN1*-affiliated clade (t3), and *SWN2*-affiliated clade (t4) were calculated and analyzed using CodeML’s free-ratio model, one-ratio model, and two-ratio model. The asterisk (**/***) indicates statistical significance at *p* < 0.05. (**c**), Divergent amino acid sites between *SWN1* and *SWN2* in the SANT, CXC, and SET domains.

To investigate the evolutionary dynamics of the *E(z)* gene, we used Fabaceae as a case study to analyze amino acid substitution rates and selection pressures. The ratio of nonsynonymous (Ka) to synonymous (Ks) substitution rates (ω = Ka/Ks) for *SWN1* and *SWN2* from *G. max*, *Medicago truncatula*, and *Phaseolus vulgaris*, with *Populus trichocarpa* as an outgroup (Figure 4a), was estimated using CodeML. Under the one-ratio model, all branches shared a uniform ω of 0.27 (Figure 4b). In contrast, the free-ratio model revealed substantial heterogeneity, with ω values ranging from 0.11 (*SWN1*, t3) to 0.63 (*SWN2*, t4). The two-ratio tests further confirmed significant lineage-specific differences: *SWN1* (t3) in *G. max* and *P. vulgaris* exhibited a markedly reduced ω (0.09, *p* = 0.0074), consistent with strong purifying selection, whereas *SWN2* (t4) in *G. max* and *P. vulgaris* displayed an elevated ω (0.84, *p* = 4.2 × 10^−5^), suggesting accelerated evolution (Figure 4b). By comparison, *SWN1* across all Fabaceae species (t1, ω = 0.28, *p* = 0.05) and *SWN2* across all Fabaceae species (t2, ω = 0.27, *p* = 0.24) did not significantly deviate from the background. Together, these results indicate that *SWN* lineages have followed divergent evolutionary trajectories, with *SWN1* constrained by strong functional conservation and *SWN2* showing evidence of rapid divergence.

To further identify amino acid sites under positive selection, we applied a branch-site model. Consistent with the branch model results, the *SWN2* clade (t2) harbored 40 amino acid sites putatively under positive selection, including 10 with posterior probabilities > 0.7 (Figure 4c and Supplementary Table S9). Mapping these sites onto functional domains revealed that most were located in the SANT (4) and SET domains (14), whereas only 2 were detected in the CXC domain (Figure 4c and Supplementary Figure S8). Previous studies have shown that the SET domain encodes histone methyltransferase (HMTase) activity [27]; and the SANT domain is composed of three alpha-helices, commonly found in proteins forming chromatin-remodeling complexes, and serves as a key region for protein–protein interactions [28]. Both the SANT and SET domains typically function in methylating lysine 9 at the N-terminus of histone H3 [29]. The occurrence of positively selected sites in these domains may therefore reflect adaptive modifications of their functions.

### 2.6. Expression Profiling of E(z) Genes in G. max

**Figure 5:**
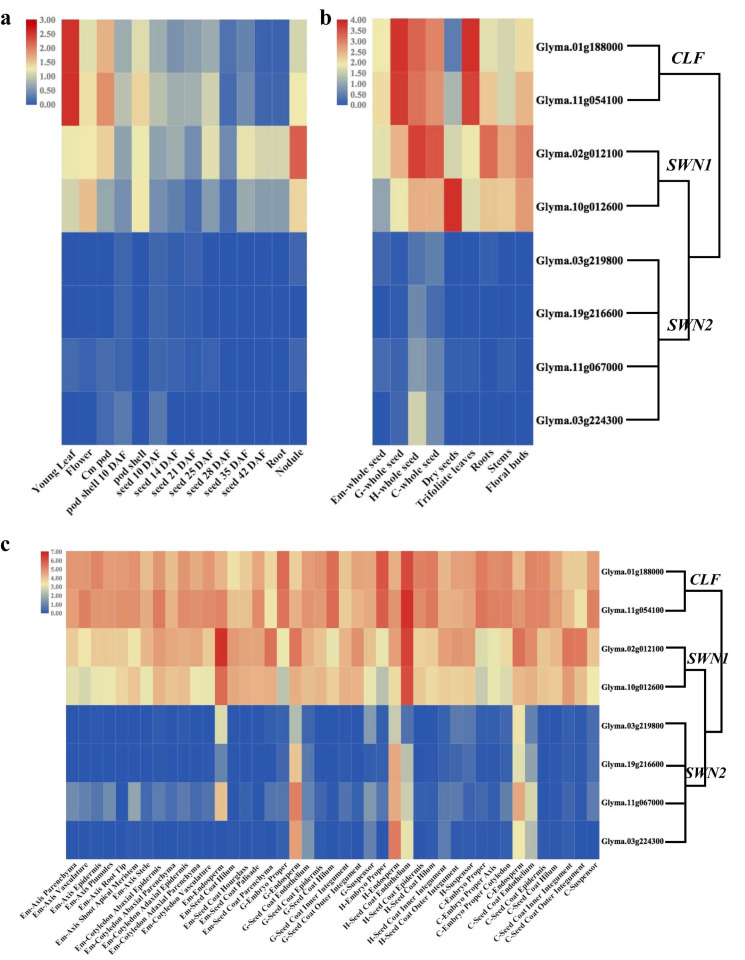
Expression patterns of *E(z)* genes in *G. max* in different tissues and developmental stages. (**a**), Heatmap of expression of *E(z)* genes in soybean tissues including young leaves, flowers, pods, husks (10 days old), husks, seeds (10 days old), seeds (14 days old), seeds (21 days old), seeds (25 days old), seeds (28 days old), seeds (35 days old), seeds (42 days old), roots, and nodules. (**b**), Expression patterns of *E(z)* genes in soybean tissues, including early-maturing seeds (Em), globular seeds (G), heart-shaped seeds (H), cotyledonary seeds (C), dry seeds, leaves, roots, stems, and flower buds. (**c**), Expression patterns of *E(z)* genes in different seed regions and tissues at four developmental stages. The horizontal axis represents different tissues and developmental stages; the vertical axis represents different genes.

To investigate how evolutionary changes may influence the function of *E(z)* genes in Fabaceae, we analyzed their temporal and spatial expression patterns. We retrieved RNA-Seq data from public databases for various tissues of *G. max*, including young leaves, flowers, pods, pod shells (10 days and later stages), seeds (14, 21, 25, 28, 35, and 45 days), roots, and nodules [30]. Based on these expression profiles, *CLF* and *SWN1* were broadly expressed across tissues (Figure 5a,b). Notably, *CLF* showed its highest expression in young leaves (Figure 5a) and in G-whole and H-whole seeds (Figure 5b), whereas *SWN1* peaked in nodules (Figure 5a) and dry seeds (Figure 5b). By contrast, *SWN2* displayed consistently low expression across most tissues, except for H-whole seeds (Figure 5b), indicating functional differentiation.

We further examined expression during seed development, including globular, heart, cotyledon, early maturation, dry seeds, trifoliate leaves, roots, stems, floral buds, and seedlings six days post-imbibition [31]. The results revealed that *SWN2* was preferentially expressed in G-endosperm, H-endosperm, and C-endosperm (Figure 5c), providing evidence for a role in endosperm development. In contrast, both *CLF* and *SWN1* displayed broader expression patterns across vegetative and reproductive organs (Figure 5c). Importantly, the expression profiles of *SWN1* and *SWN2* in *G. max* resembled those of *SWN* and *MEA* in Arabidopsis [32], respectively, suggesting that Fabaceae *SWN2* may play a role similar to the imprinted *MEA* gene in Brassicaceae during seed development.

## 3. Discussion

### 3.1. Conservation and Divergence of E(z) Genes in Green Plants

In this study, we conducted a phylogenetic analysis of 431 *E(z)* genes from 193 green plant species. We found that *E(z)* homologs are present as single copies in early land plants, indicating highly conserved functions in these lineages (Figure 1a,c). By contrast, in seed plants, *E(z)* homologs underwent duplications that gave rise to two distinct clades: *CLF* and *SWN*. These two clades are generally maintained as single or low copy numbers across most lineages, with notable lineage-specific expansions observed in some seed plants.

The most pronounced expansions occurred in angiosperms, particularly in Fabaceae and Brassicaceae. Despite the overall conservation (11 out of 20 shared motifs), the *CLF* and *SWN* clades display markedly different motif patterns (Figure 3a). In the *CLF* clade, motif patterns of gymnosperms and angiosperms are nearly identical, suggesting strong functional conservation. By contrast, in the *SWN* clade, gymnosperms retain motifs 14 and 19, which are absent in angiosperms except for basal lineages (Supplementary Figure S1). Moreover, the *SWN* clade exhibits larger Ka/Ks ratios (ω = 0.51) compared with the CLF clade (ω = 0.06) (Supplementary Figure S2), indicating that *SWN* genes are subject to stronger positive selection. These results suggest that while *CLF* has remained evolutionarily constrained and functionally stable, *SWN* has undergone greater evolutionary flexibility, contributing to functional divergence in seed plants.

### 3.2. Neo-Functionalization of E(z) in Fabaceae

*E(z)* genes experienced two major rounds of duplication during green plant evolution. The first duplication occurred before the divergence of angiosperms and gymnosperms, giving rise to the *CLF* and *SWN* clades. A second round of whole-genome duplication (WGD) in Brassicaceae and Fabaceae subsequently produced additional subclades: *SWN* and *MEA* in Brassicaceae [26] and *SWN1* and *SWN2* in Fabaceae. The divergence of these subclades is further supported by structural modifications, including the loss of ~200 amino acids in the Fabaceae *SWN2* lineage, accompanied by distinct exon–intron structures (Supplementary Figure S5).

Molecular evolutionary analyses revealed heterogeneous selective pressures between *SWN1* and *SWN2*. Under the free-ratio model, ω values varied considerably, ranging from 0.11 (*SWN1*, t3) to 0.63 (*SWN2*, t4). Two-ratio tests confirmed significant lineage-specific differences: *SWN1* (t3) in *G. max* and *P. vulgaris* exhibited a markedly reduced ω (0.09, *p* = 0.0074), consistent with strong purifying selection, whereas *SWN2* (t4) showed an elevated ω (0.84, *p* = 4.2 × 10^−5^), suggesting accelerated evolution. Branch-site tests further identified 40 amino acid sites putatively under positive selection in *SWN2*, including 10 with posterior probabilities > 0.7 (Figure 4c; Supplementary Table S9). Notably, several of these sites were located within the SANT and SET domains, providing strong evidence for functional diversification within Fabaceae.

Expression profiling in *G. max* revealed that *SWN2* is preferentially expressed in reproductive tissues, particularly in whole seeds and endosperm, whereas *SWN1* maintains broader expression across tissues. This complementary pattern indicates that *SWN2* is undergoing neo-functionalization, potentially specializing in seed and endosperm regulation [26]. Given its parallels to the imprinted *MEA* gene in Arabidopsis, *SWN2* may represent a Fabaceae-specific candidate for imprinted regulation in seed development.

Taken together, our findings provide new insights into the evolutionary history and functional diversification of *E(z)* homologs across 271 green plants. In particular, we highlight how lineage-specific duplication and divergence in Fabaceae have driven the emergence of specialized regulatory roles, offering a framework for understanding how chromatin regulators evolve to shape developmental processes in plants.

## 4. Materials and Methods

### 4.1. Data Collection

The *E(z)* genes (*AT1G02580*, *AT4G02020*, *AT2G23380*) from TAIR [33] (http://www.arabidopsis.org, accessed on 5 November 2025) were used as the initial queries. Protein and coding sequences of green plants were obtained from public databases, including Phytozome v13.0 [34] (https://phytozome.jgi.doe.gov/, accessed on 5 November 2025), CoGe [35] (https://genomevolution.org/coge/index.pl, accessed on 5 November 2025), OneKP [36] (1000 Plants; https://www.onekp.com, accessed on 5 November 2025), GigaDB [37] (http://gigadb.org/, accessed on 5 November 2025), and PlantGIR [38] (http://plantgir.cn/). A total of 193 plant species were included.

### 4.2. Identification of E(z) Homolog Genes

The *E(z)* genes from TAIR were aligned against the 193 collected plants gene set using blastp [39] (E-value < 1 × 10^−5^) and HMMER [40] (E-value < 1× 10^−5^). Only candidate sequences identified by both methods were retained. To improve sequence quality, several filtering steps were applied. First, short sequences (<150 amino acids) were removed, as they could negatively impact subsequent alignments and phylogenetic tree construction. Second, when two or more protein sequences were highly similar and overlapped at the same locus, the longest protein sequence was selected. The retained sequences were then subjected to domain analysis using online resources including Pfam [41] (hosted by InterPro https://www.ebi.ac.uk/interpro/entry/pfam/, accessed on 5 November 2025), NCBI CDD [42] (http://www.ncbi.nlm.nih.gov/cdd/, accessed on 5 November 2025), and SMART [43]) (http://smart.embl-heidelberg.de, accessed on 5 November 2025), with default parameters. Sequences lacking a recognizable SET domain were excluded. The final set of Final E(z) protein sequences has been deposited in Zenodo repository (https://zenodo.org/records/17445749, accessed on 5 November 2025).

### 4.3. Phylogenetic Analysis

We applied a consistent pipeline for phylogenetic analyses throughout this study. Protein sequences of *E(z)* genes were first aligned using MUSCLE v3.8.31 [44], which provides high accuracy and computational efficiency for multiple sequence alignment. The alignments were then trimmed using using trimAl v1.4.1 [45] (-gt 0.8 -st 0.001 -cons 80) to remove poorly aligned or divergent regions, thereby improving the reliability of downstream analyses. The alignment file was provided in Zenodo (https://zenodo.org/records/17445749, accessed on 5 November 2025). Maximum likelihood (ML) trees were constructed with FastTree v2.1 [46] (parameter: -gtr) which is optimized for large-scale datasets and offers a balance of speed and accuracy in tree inference. Detailed methods were also provided in the Zenodo repository. The resulting gene trees were visualized and annotated using iTOL v6 [47], allowing for interactive exploration and clear presentation of phylogenetic relationships.

### 4.4. Motif Patterns and Gene Structure

All amino acid sequences from selected species and Brassicaceae and Fabaceae were searched against the Pfam (https://www.ebi.ac.uk/interpro/entry/pfam/, accessed on 5 November 2025) and CDD (http://www.ncbi.nlm.nih.gov/cdd/, accessed on 5 November 2025) databases to identify conserved motifs. Additionally, to discover novel conserved motifs not recorded in public databases, we used the Multiple Em for Motif Elicitation (MEME) v4.9.0 software [48] (parameter: minimum motif width 10 aa, maximum motif width 80 aa, minimum number of sites 20). The conserved motif patterns were visualized and redrawn using TBtools v2.2 [49]. The presence of introns and exons was annotated based on the *A. thaliana* and *G. max* annotation files from Phytozome. Gene structure diagrams were generated using GSDS v2 [50].

### 4.5. Synteny and Selection Analysis

Genome synteny between SWN1 and SWN2 from *G.max* was analyzed using the Plant Genome Duplication Database (PGDD) [51] (http://chibba.agtec.uga.edu, accessed on 5 November 2025), as it provides comprehensive data on gene duplications and syntenic relationships across plant genomes, enabling accurate identification of conserved genomic regions.

To investigate variations in selective pressures and identify positively selected sites in representative plants (Figure 1), the CodeML program in the PAML package [52] was used to calculate the ω (dN/dS) ratio, which quantifies the rate of non-synonymous to synonymous substitutions. To evaluate selection variation across Fabaceae, branch model and branch-site model tests were conducted using the EasyCodeML v1 [53], chosen for its user-friendly interface and robust statistical framework to detect positive selection sites in specific clades (t1, t2, t3, and t4). Three site models in EasyCodeML were explored to identify site-specific selection patterns, providing a comprehensive analysis of evolutionary pressures.

### 4.6. Expression Analysis

To determine the expression patterns of the E(z) homolog genes (Glyma.03G219800, Glyma.19G216600, Glyma.11G067000, Glyma.03G224300, Glyma.02G012100, Glyma.10G012600, Glyma.01G188000, Glyma.11G054100) in G. max tissues, transcriptome data were obtained from public websites. The six transcriptome data sets included RNA-seq dataset 1-6 (accession number: PMC3017786 [30], PMC2815892 [54], GSE46096 (https://www.ncbi.nlm.nih.gov/geo/query/acc.cgi?acc=GSE46096, accessed on 5 November 2025), GSE57349 (https://www.ncbi.nlm.nih.gov/geo/query/acc.cgi?acc=GSE57349, accessed on 5 November 2025), GSE57350 (https://www.ncbi.nlm.nih.gov/geo/query/acc.cgi?acc=GSE57350, accessed on 5 November 2025), GSE57606 (https://www.ncbi.nlm.nih.gov/geo/query/acc.cgi?acc=GSE57606, accessed on 5 November 2025). RNA-seq dataset 1 identified expression patterns in different soybean tissues, including young leaves, flowers, pods, husks (10 days), husks, seeds (10 days), seeds (14 days), seeds (21 days), seeds (25 days), seeds (28 days), seeds (35 days), seeds (42 days), roots, and nodules. RNA-seq dataset 2 analyzed expression patterns in soybean seeds at different developmental stages, including early-maturing seeds, globular seeds, heart-shaped seeds, cotyledonary seeds, dry seeds, leaves, roots, stems, and flower buds. We further analyzed the expression levels of different seed parts during four seed developmental stages: early-maturing seeds, globular seeds, heart-shaped seeds, and cotyledonary seeds using RNA-seq datasets 3-6. The FPKM values of target genes were collected and merged into one table using R v4.3.2 (https://www.r-project.org/, accessed on 5 November 2025), then scaled and plotted using TBtools.

## Data Availability

The data presented in this study are openly available in https://zenodo.org/records/17445749 (accessed on 5 November 2025).

## Supplementary Materials

The following supporting information can be downloaded at: https://www.mdpi.com/article/10.3390/plants14223444/s1, Figure S1: Analysis of conserved domains and motifs of E(z) proteins from representative species of various plant groups; Figure S2: Selection analysis of *CLF *and *SWN*; Figure S3: Genome synteny between *SWN1 *and *SWN2 *in *G.max*; Figure S4: The 20 motifs were identified in Brassicaceae and Fabaceae, named Motif 1-20; Figure S5: The exon/intron structures of *E(z)* homologous genes; Figure S6: The relationship of several motifs and exons; Figure S7: Phylogenetic analysis and conserved domains of the *E(z)* homologous genes in Brassicaceae and Fabaceae; Figure S8: Multiple sequence alignment with differentiated amino acid sites between *SWN1 *and *SWN2 *in Fabaceae; Table S1: Statistics of *E(z)* homologues in Eudicots; Table S2: Statistics of *E(z)* homologues in Monocots; Table S3: Statistics of *E(z)* homologues in Basal angiosperm; Table S4: Statistics of *E(z)* homologues in Gymnosperm; Table S5: Statistics of *E(z)* homologues in Lycophytes; Table S6: Statistics of *E(z)* homologues in Bryophytes; Table S7: Species with gene numbers used for Seed plant *E(z)* phylogeny analysis; Table S8: Species with gene numbers used for Angiosperm *E(z)* phylogeny analysis; Table S9: Branch-site model analysis for SWN amino acid changes in *G. max*.
