# Supplementary material for: Conservation and Divergence of E(z) Genes in Green Plants
Source: Plants (Basel). 2025 Nov 11;14(22):3444. doi: 10.3390/plants14223444 (PMC12655823; doi:10.3390/plants14223444)
Supplement: Supplementary file 1 [file plants-14-03444-s001.zip › Supplementary_Figures.pdf]

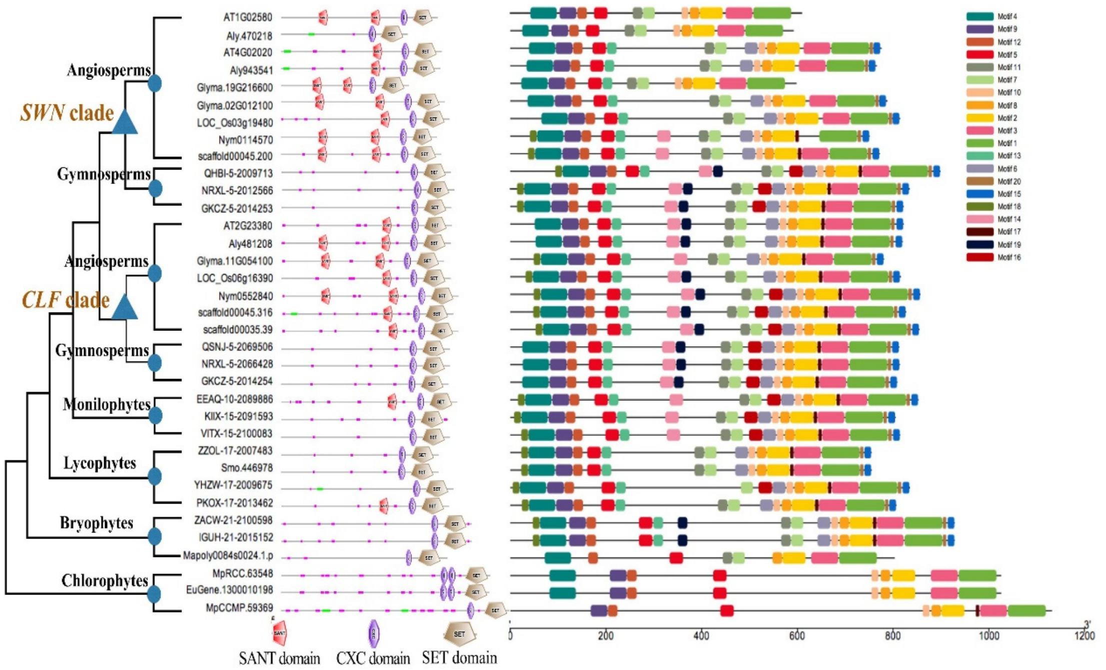

Supplementary Figure S1 Analysis of conserved domains and motifs of E(z) proteins from representative species of various plant groups.

Note: The schematic diagram constructed based on the topology of the phylogenetic tree of green plants (branches marked with blue circles represent various plant groups, and branches marked with blue triangles represent the *CLF* and *SWN* clades). A total of three conserved domains were identified. Conserved motifs corresponding to E(z) proteins from representative species (a total of 20 conserved motifs were identified, designated Motifs 1–20).

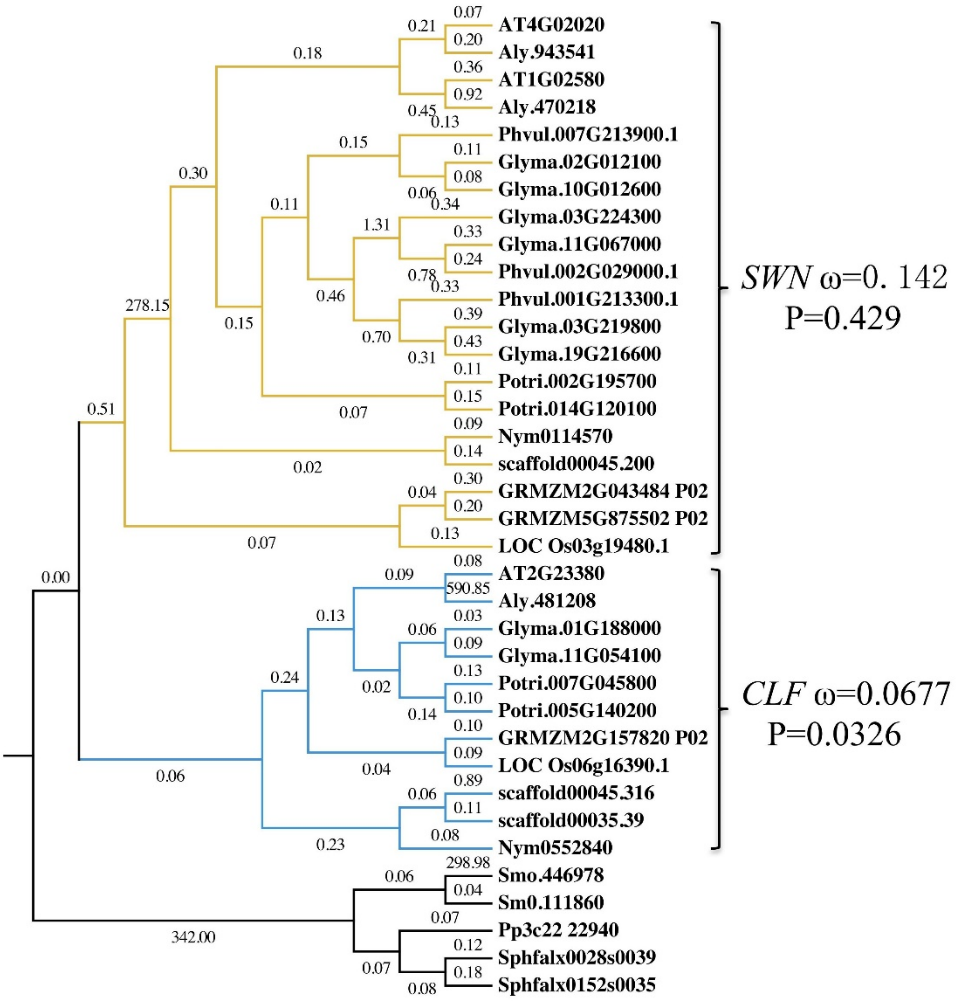

Supplementary Figure S2 Selection analysis of *CLF* and *SWN.*

Note: The ML phylogenetic tree was constructed using the native FastTree program. The average Ka/Ks values for each clade were then calculated using the PAML software based on the phylogenetic tree.

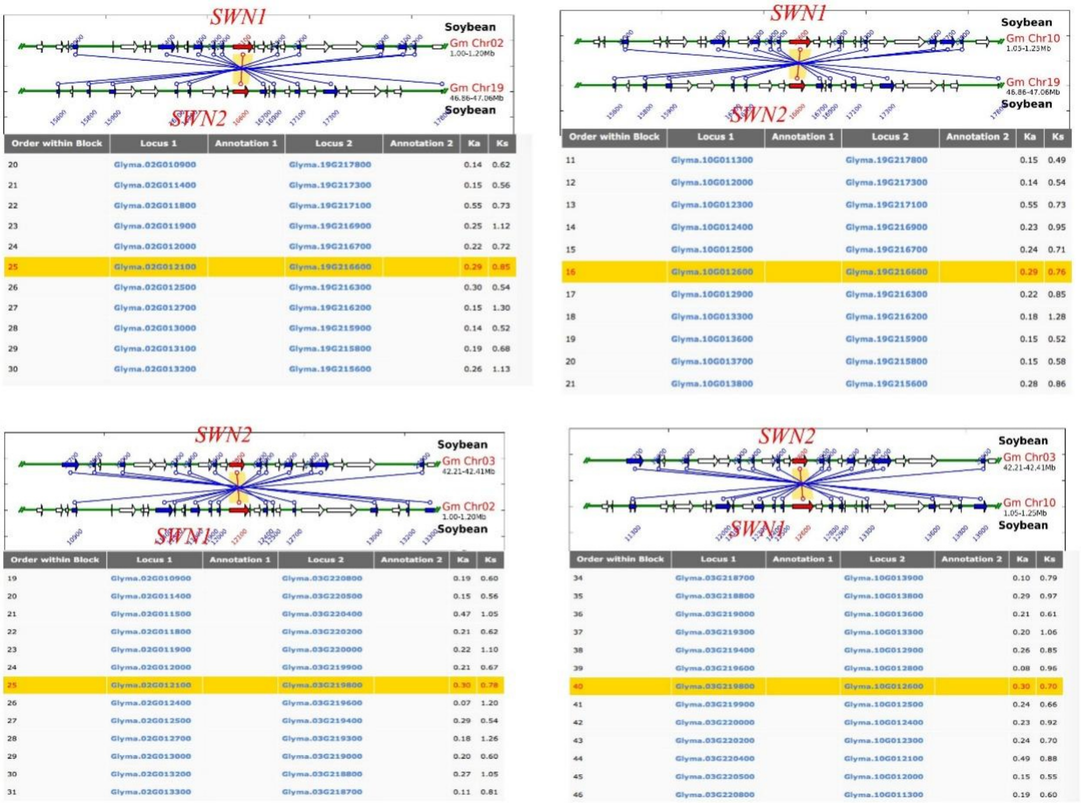

Order within Block	Locus 1	Annotation 1	Locus 2	Annotation 2	Ka	Ks
20	Glyma.02G010900		Glyma.19G217800		0.14	0.62
21	Glyma.02G011400		Glyma.19G217300		0.15	0.56
22	Glyma.02G011800		Glyma.19G217100		0.55	0.73
23	Glyma.02G011900		Glyma.19G216900		0.25	1.12
24	Glyma.02G012000		Glyma.19G216700		0.22	0.72
25	Glyma.02G012100		Glyma.19G216600		0.29	0.85
26	Glyma.02G012500		Glyma.19G216300		0.30	0.54
27	Glyma.02G012700		Glyma.19G216200		0.15	1.30
28	Glyma.02G013000		Glyma.19G215900		0.14	0.52
29	Glyma.02G013100		Glyma.19G215800		0.19	0.68
30	Glyma.02G013200		Glyma.19G215600		0.26	1.13

Order within Block	Locus 1	Annotation 1	Locus 2	Annotation 2	Ka	Ks
11	Glyma.10G011300		Glyma.19G217800		0.15	0.49
12	Glyma.10G012000		Glyma.19G217300		0.14	0.54
13	Glyma.10G012300		Glyma.19G217100		0.55	0.73
14	Glyma.10G012400		Glyma.19G216900		0.23	0.95
15	Glyma.10G012500		Glyma.19G216700		0.24	0.71
16	Glyma.10G012600		Glyma.19G216600		0.29	0.76
17	Glyma.10G012900		Glyma.19G216300		0.22	0.85
18	Glyma.10G013300		Glyma.19G216200		0.18	1.28
19	Glyma.10G013600		Glyma.19G215900		0.15	0.52
20	Glyma.10G013700		Glyma.19G215800		0.15	0.58
21	Glyma.10G013800		Glyma.19G215600		0.28	0.86

Order within Block	Locus 1	Annotation 1	Locus 2	Annotation 2	Ka	Ks
19	Glyma.02G010900		Glyma.03G220800		0.19	0.60
20	Glyma.02G011400		Glyma.03G220500		0.15	0.56
21	Glyma.02G011500		Glyma.03G220400		0.47	1.05
22	Glyma.02G011800		Glyma.03G220200		0.21	0.62
23	Glyma.02G011900		Glyma.03G220000		0.22	1.10
24	Glyma.02G012000		Glyma.03G219900		0.21	0.67
25	Glyma.02G012100		Glyma.03G219800		0.30	0.78
26	Glyma.02G012400		Glyma.03G219600		0.07	1.20
27	Glyma.02G012500		Glyma.03G219400		0.29	0.54
28	Glyma.02G012700		Glyma.03G219300		0.18	1.26
29	Glyma.02G013000		Glyma.03G219000		0.20	0.60
30	Glyma.02G013200		Glyma.03G218800		0.27	1.05
31	Glyma.02G013300		Glyma.03G218700		0.11	0.81

Order within Block	Locus 1	Annotation 1	Locus 2	Annotation 2	Ka	Ks
34	Glyma.03G218700		Glyma.10G013900		0.10	0.79
35	Glyma.03G218800		Glyma.10G013800		0.29	0.97
36	Glyma.03G219000		Glyma.10G013600		0.21	0.61
37	Glyma.03G219300		Glyma.10G013300		0.20	1.06
38	Glyma.03G219400		Glyma.10G012900		0.26	0.85
39	Glyma.03G219600		Glyma.10G012800		0.08	0.96
40	Glyma.03G219800		Glyma.10G012600		0.30	0.70
41	Glyma.03G219900		Glyma.10G012500		0.24	0.66
42	Glyma.03G220000		Glyma.10G012400		0.23	0.92
43	Glyma.03G220200		Glyma.10G012300		0.24	0.70
44	Glyma.03G220400		Glyma.10G012100		0.49	0.88
45	Glyma.03G220500		Glyma.10G012000		0.15	0.55
46	Glyma.03G220800		Glyma.10G011300		0.19	0.60

Supplementary Figure S3 Genome synteny between *SWN1* and *SWN2* in *G.max*.

Note: *Glyma.02G012100* (*SWN1*) VS *Glyma.19G216600* (*SWN2*);

Glyma.10G012600 (SWN1) VS *Glyma.19G216600* (*SWN2*);

Glyma.02G012100 (SWN1) VS *Glyma.03G219800* (*SWN2*);

Glyma.03G219800 (SWN2) VS *Glyma.10G012600* (*SWN1*)

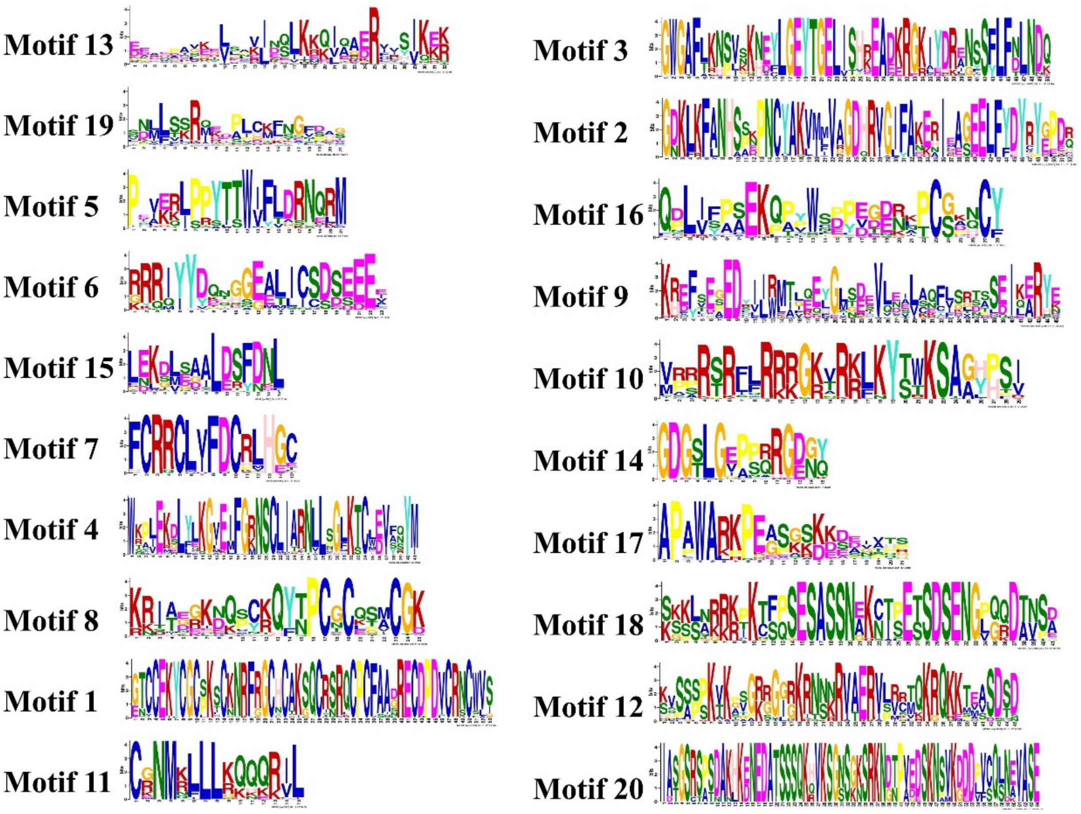

Supplementary Figure S4 The 20 motifs were identified in Brassicaceae and Fabaceae, named Motif 1-20.

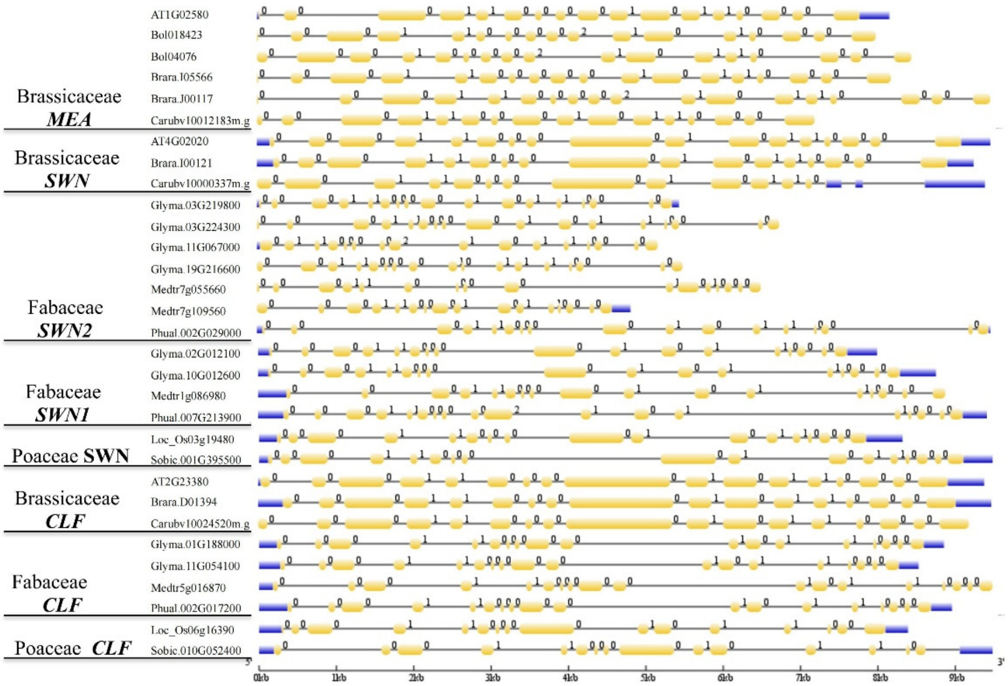

Gene \ Exon		1	2	3	4	5	6	7	8	9	10	11	12	13	14	15	16	17
MEA	AT1G02580	9	90	332	174	88	126	48	69	149	127	209	132	82	48	129	78	180
SWN	AT4G02020	36	120	266	159	85	144	48	72	605	148	221	132	88	48	129	78	192
CLF	AT2G23380	63	93	302	162	85	135	48	72	701	151	224	132	91	48	129	78	195
MEA	Glyma.11G067000			218	162	85	162	48	72	94 208	148	221	132	88	48	129	78	210
	Glyma.19G216600		105	269	162	85	162	48	72	146	148	56 123	132	88	48	129	78	186
	Glyma.03G224300	39	105	269	162	85	162	48	72	461	148	221	132	88	48	129	78	198
	Glyma.03G219800	39	105	269	162	85	162	48	72	254	148	218	132	88	48	129	78	198
SWN	Glyma.02G012100	39	102	272	162	85	156	48	72	629	154	221	132	88	48	129	78	192
	Glyma.10G012600	39	102	272	162	85	156	48	72	635	154	221	132	88	48	129	78	192
CLF	Glyma.01G188000	69	102	326	162	85	156	48	72	345 191	136	224	132	91	48	129	78	192
	Glyma.11G054100	69	102	326	162	85	156	48	72	345 191	136	224	132	91	48	129	78	192

Supplementary Figure S5 The exon/intron structures of *E(z)* homologous genes.

Note: Top panel showed *E(z)* homologous genes structure in Brassicaceae, Fabaceae and Poaceae. Bottom panel showed the exon length and exon number of *E(z)* homologous genes in *G. max* and *A. thaliana*

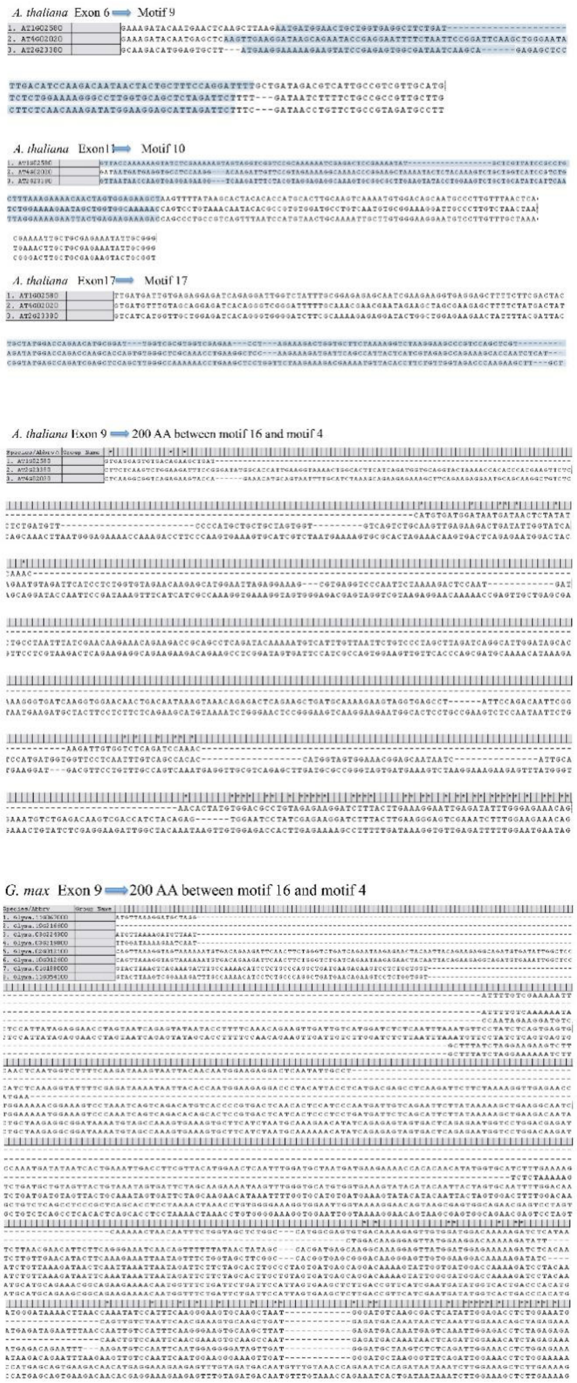

Supplementary Figure S6 The relationship of several motifs and exons.

Note: Top panel: Arabidopsis Motif 9, Motif 10, and Motif 17 correspond to exons 6, 11, and 17, respectively. The approximately 200-AA deletion between *A. thaliana* Motif 16 and Motif 4 corresponds to exon 9. Bottom panel: the approximately 200-AA deletion between *G.max* Motif 16 and Motif 4.

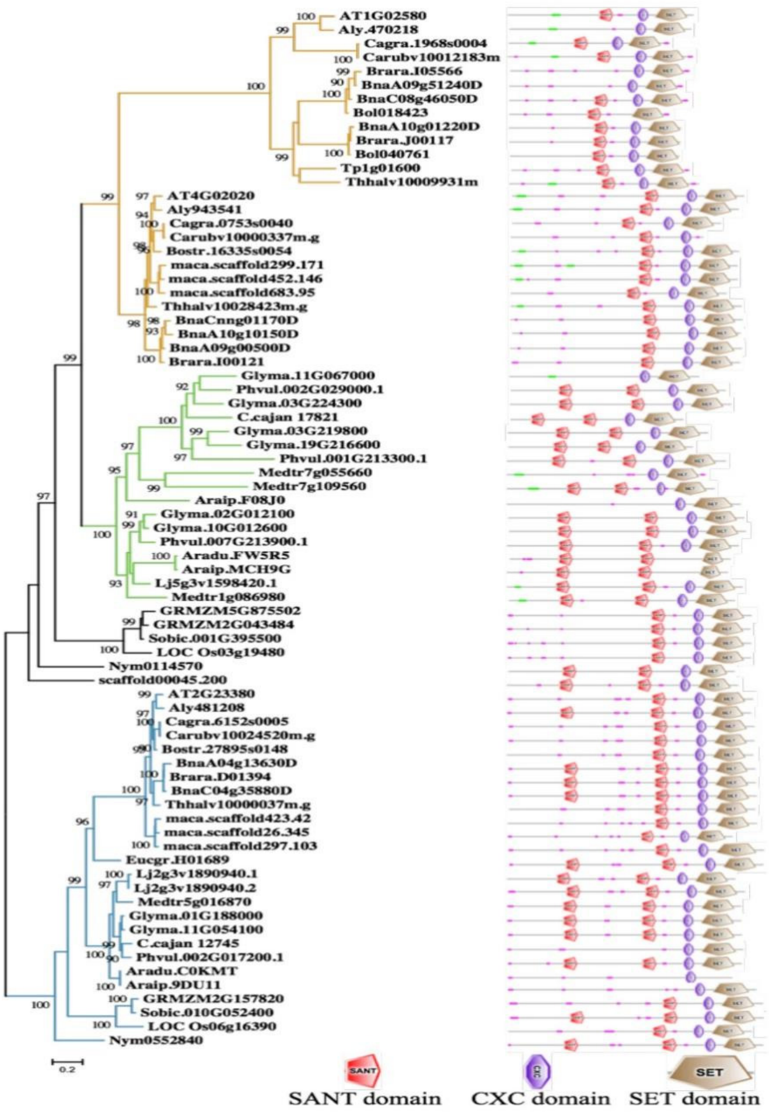

Supplementary Figure S7 Phylogenetic analysis and conserved domains of the *E(z)* homologous genes in Brassicaceae and Fabaceae.

Note: Phylogenetic tree of *E(z)* homologous genes in Brassicaceae and Fabaceae (yellow branches represent Brassicaceae in the *SWN* clade, green branches represent Fabaceae in the *SWN* clade, and blue branches represent the *CLF* clade). Conserved domain pattern diagram displayed using online SMART software (different shapes represent different domains; a total of three conserved domains were identified).

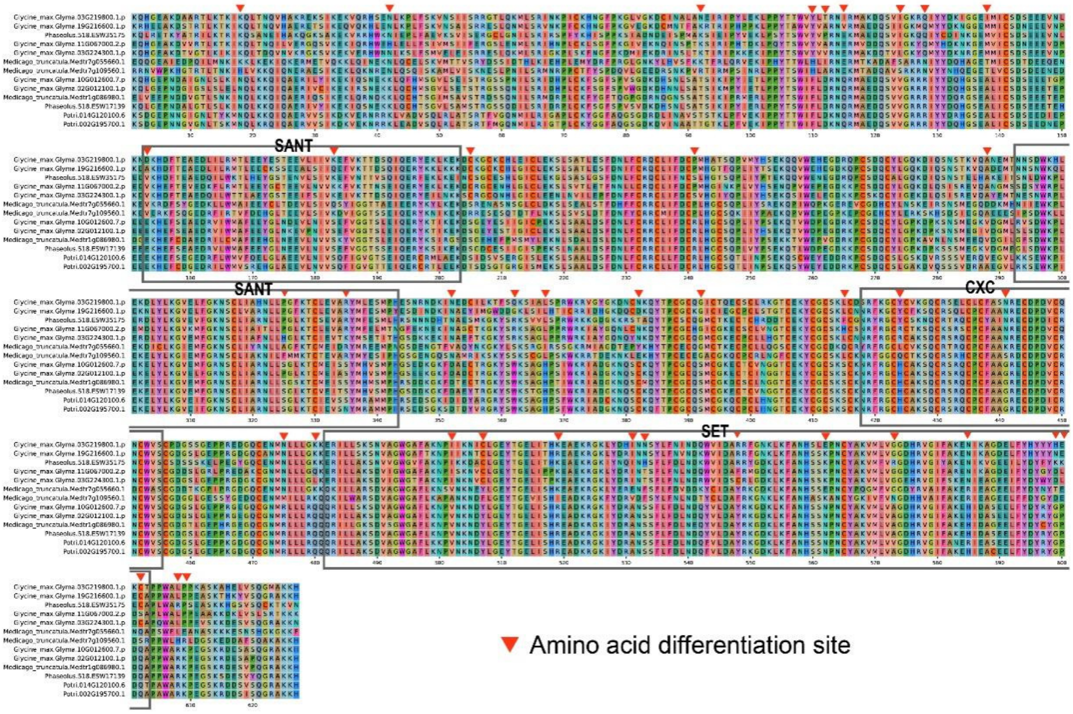

Supplementary Figure S8 Multiple sequence alignment with differentiated amino acid sites between *SWN1* and *SWN2* in Fabaceae.
